# Genotoxicity  effects in Cancer Patients: Evaluation by Micronucleus and Comet Assays and Correlation with Biochemical and Hematological Indices

**DOI:** 10.12688/f1000research.178233.2

**Published:** 2026-07-17

**Authors:** Sarab Dalaf Khalaf, Atyaf Talal Mahmood, Alyaa Fadhil Abbas, Ruwaida Watheq Neamah, Rana Nasser Abdul Hameed

**Affiliations:** 1 Biology, Tikrit University College of Science, Tikrit, Saladin Governorate, Iraq; 2 university of Mosul college of pharmacy, mosul, Iraq; 3 Salah ad-Din Education Directorate, Tikrit, Iraq; 4 Salahaddin oncology center (SOC), Tikrit, Iraq; 5 Northern Technical University /Institute Al-Dour Technical, Tikrit, Iraq

**Keywords:** Keywords: Cancer, Buccal Micronucleus Assay, Comet Assay, Genomic Instability, Hematological Parameters, biochemical parameters, carcinoma, DNA .

## Abstract

**Background:**

Cancer is one of the most significant global health challenges. Cytogenetic techniques are used as biomarkers of cancer risk to detect DNA damage and chromosomal abnormalities.

**Objective:**

This study aimed to evaluate the influence of chemotherapy on genetic stability in cancer patients by applying Micronucleus and Comet assays as indicators of DNA and chromosomal alterations. It also aimed to determine the possible links between these genotoxic changes and variations in biochemical and hematological parameters, providing insight into the cellular responses to chemotherapy exposure.

**Methods:**

Buccal epithelial cells were collected from 190 individuals (90 controls and 100 cancer patients) and stained for micronucleus analysis. To observe DNA breakdown, cells from blood were subjected to an alkaline comet assay. Biochemical (urea, creatinine, calcium, and bilirubin) and hematopoietic (Hb, WBC, Plt, and HCT) parameters were also assessed.

**Results:**

Cancer patients showed elevated Binucleated cell (27.50 ± 3.62),Condensed chromatin (10.50 ± 2.87), broken eggs (2.65 ± 1.53), basal cell (4.65 ± 1.98), Karyorrhetic cell (9.45 ± 1.73), Karyolytic cell(85.10 ± 2.69), Monoonucleated cell with Micronucleus(12.50 ± 1.43), and Percentage of nuclear anomalies in total(152.8 ± 4.75) compared with controls. The comet assay results revealed pronounced DNA fragmentation in cancer cells, with variable tail lengths indicating heterogeneous damage. The biochemical results showed a significant increase in the mean levels of urea (50.600 ± 8.259) and creatinine (1.073 ± 0.173), and no significant increase in TSB (0.917 ± 0.143) and HCT(38.080 ± 2.311) in cancer patients compared to the control. Calcium (9.010 ± 0.680), Hb (8.322 ± 1.247), Plt (91.300 ± 24.495), and WBC (11.920 ± 2.833) levels were lower in the patient group than in the control group.

**Conclusion:**

The increased frequency of nuclear anomalies and DNA fragmentation in cancer patients highlights the potential of the buccal Micronucleus assay and comet assays as effective, non-invasive tools for early cancer detection and genotoxic monitoring. Alterations in the blood and biochemical parameters further support the systemic effects of malignancy.

## Introduction

Carcinoma is a serious medical issue among the primary causes of death worldwide. Its classification varies according to the tissue from which it arises and the organs in which the disease develops. ^1^ The type of tumor is determined by microscopic examination of the tumor cells, which is sometimes supported by blood. It is usually difficult to determine the cause of the disease; however, we know the general causes of cancer. Many risk factors can cause this disease, such as obesity, lack of physical activity, smoking, and environmental pollution. ^2^ Although this disease is considered fatal, recovery from it is continually increasing in the majority of cases as a result of improvements in therapeutic options as well as early diagnosis techniques. ^3^

Investigation suggests that buccal cell microscopic nuclei (MNs) are significantly elevated in the buccal mucous membrane cells of people with precancerous tumors and those with cancer, making them possible biomarkers for cancer of the mouth. ^4^ Chromosome fragmentation from damaged or disorganized chromosomes or inadequately repaired DNA lesions resulting from mitotic mistakes causes micronuclei to develop in proliferating cells. ^5^ Deficits in micronutrients are needed as cofactors for DNA disintegration and chromosomal separation, oxidative damage, aneugen exposure to clastogens, genetic anomalies in DNA repair genes and/or cell cycle checkpoints, and more can all contribute to these occurrences. ^6^ MN develops as a result of chromosomal rearrangements, modifications in the expression of genes, aneuploidy, and outcomes related to chromosomal instability frequently observed in cancer. ^7^ Genetic damage is the only factor that the chromosomal aberration test can detect; however mitotic spindle malfunction and chromosome loss due to aneugenic processes can also be detected by the micronucleus test. ^8^ One theory suggests that micronuclei and chromosomal aberrations may possess predictive value for cancer, perhaps serving as substitutes for chromosomal aberrations as indicators of cancer risk or offering additional insights into the mechanisms of aneugenic agents. ^9^ For assessing genetic damage in people, the MN test of buccal exfoliation is a potentially useful and somewhat easy technique. ^10^ In addition to micronuclei (MNi), this assay can also detect other nuclear aberrations resulting from both genotoxic and cytotoxic effects. ^11^ In addition to MNi, buccal cells can also exhibit nuclear abnormalities such as BN, Karyorrhexis (KR), pyknotic nuclei (P), and karyolysis (KL). ^12^ Binucleated BN is an instance of spindle disruption (aneugenic repercussions), whereas MNi and broken eggs (BE) are instances of genotoxic occurrences. Pyknosis, concentrated chromatin (CC), KR, and KL are examples of acute cytotoxic effects. ^9^ According to their examination of all the information regarding the MN experiment in the buccal cells of patients with cancer. ^13^ concluded that the occurrence of MN and other endpoints significantly increases with cancer diagnosis. For malignancies of the respiratory system, oropharyngeal tumors, and all other cancers combined, a very strong connection was found.

### Aim of study

The main goal of this investigation was to determine the incidence of nuclear anomalies, including micronuclei, in mucosal cells extracted from cancer patients.

### Objectives of this study

The objective of this study was to quantify DNA damage in cancer patients and identify the presence of micronuclei and other nuclear abnormalities in excised buccal cells of the mouth by combining hematologic and biochemical surveillance with a comet impact assay.

### Sample collection and preparation

Whole blood samples were collected from both cancer patients and the control group between 2/12/2023 and 20/5/2024. Approximately 5 mL of venous blood was drawn from each participant using sterile disposable syringes. The collected blood was immediately divided into two portions:

Portion 1: Transferred into EDTA-coated tubes for immediate analysis of hematological indices. A part of this portion was also processed directly for the Comet assay (Single Cell Gel Electrophoresis) and stored at −20 °C for future Comet assay analysis.

Portion 2: Transferred into heparinized tubes for biochemical analysis, which was performed directly on the same day of collection.

All samples were maintained at 4 °C in a cooling box during transportation and were processed within 4 hours of collection to ensure cell viability and prevent DNA degradation. Samples designated for the Comet assay that were not processed immediately were stored at −20 °C until analysis.

## Methods

### Study design

A total of 190 samples (45–60 age) were collected from random people with and without cancer, 90 from healthy people (without cancer), and 100 from people with different types of cancer (prostate cancer, breast cancer, colon cancer, lymphatic gland cancer, and lung cancer). Histopathological diagnoses were confirmed by the oncology center for each patient. Sample were collected from buccal cells for micronucleus assay, and the blood for comet assay, and other biological indices.

### Assay for micronuclei in exfoliated buccal cells

The investigation was conducted as follows, following the steps described by Gopal and Padma ^14^: following the instructions for washing their salivary glands with water, the participants collected cells from the mucous membrane of their mouths using a wooden spatula that had been previously moistened, and subsequently placed on a clean microscope slide. They were air-dried and preserved in methyl alcohol (96%) for 3–5 min. The specimen was stained with May-Grunwald stain for 20–25 min, followed by Gemza staining for 3–5 min, wished, allowed to dry, and examined under a microscope (40x). The incidence of micronuclei was documented, with 2000 cells assessed for each subject.

### Alkaline comet assay

During this test, a treatment technique (Trevigen, Inc., Gaithersburg, US) was applied. The Lysis Solution was prepared and then allowed to cool for 20 min. Add cells with 1 × 10 ^5^/ml melted low point of melting (LMA) after 50 μl is dispensed onto a Comet SlideTM. Agarose. Agarose and cells were evenly distributed throughout the sample using a pipette tip. After being flattened, the microscope slides were placed in the dark environment and cooled for 10 min at 4 °C. To improve sample adherence in heavy humidity, the gelling time might be prolonged to 30 min. After 30 to 60 min, the slides were removed out of a 4 °C Lysis Solution to get rid of any remaining buffer that Com-. For one hour, a freshly made Alkaline Relaxation Solution from the company—which had a pH higher than 13—was submerged at 4 °C. The slides were placed in a tray with labels toward the black cathode after the alkaline electrophoresis solutions were added. The power was switched on for 30 min at a voltage of 21 volts. The samples were carefully immersed twice in 70% ethanol for five minutes, twice in distilled water, and twice in electrophoresis solution. The materials were examined after being dehydration at 37 °C for 10 to 15 minutes. After applying 1X Ethidium Bromide to the Comet Slide TM slide to eliminate discoloration, cold distilled water was used for rinsing. The slides were quickly assessed. A fluorescent microscope with a 40X lens plus a touchscreen camera enabling slide processing was used to measure the DNA damage. Four comet images were evaluated using 50 randomly selected cells, accounting for 25 cells per plate. We examined the number and rate of migration of the damaged cells. ^15^

### Study of some biochemical and hematological parameters

Urea, creatinine, calcium, and total serum bilirubin levels were estimated using the COBAS C111 device, while the Converges device was used for complete blood count.

### Data analysis

A t-test was used to analyze the data. Duncan’s original varied range test was used to minimize the mean difference as the mean ± SD. SPSS (version 28.0, SPSS Ltd., Surrey, UK) was used for each statistical analysis.

### Ethical considerations and informed consent

Ethical Approval: This study was conducted in accordance with the ethical principles of the Declaration of Helsinki. The research protocol was reviewed and approved by the Pharmaceutical Research Ethics Committee (PREC) at the University of Mosul, College of Pharmacy (Reference Code: PREC-25-3-10; Date: October 12, 2025).

**Note:** The title mentioned in the ethical certificate [Ref: PREC-25-3-10] represents the initial study title; the current manuscript title is an updated version of the same study.

**Informed Consent**: Written informed consent was obtained from all individual participants included in the study. All participants were fully briefed on the study’s objectives, the blood collection procedure, and their right to withdraw at any time. Confidentiality of all personal and medical data was strictly maintained throughout the study.

## Results

### Assessment of micronuclei in oral lining epithelial cells in cancer patients

Identifying indications of early biological impacts is the aim of examining micronuclei in buccal epithelial cells. The frequency of micronuclei in buccal epithelial cells was determined by producing violet-colored samples and examining the outcomes under a 40X magnification microscope. As shown in Table 1, all micronucleus parameters were significantly higher in cancer patients compared to the control group (p < 0.001).

**Table 1.** Frequency of micronucleus abnormalities in buccal epithelial cells of control and cancer patient groups.

Parameter	Control mean ± SD	Patient mean ± SD	p-value
Binucleated cell	4.05 ± 1.50	27.50 ± 3.62	< 0.001
Condensed chromatin	8.40 ± 2.03	10.50 ± 2.87	< 0.001
Broken eggs	1.70 ± 1.17	2.65 ± 1.53	< 0.001
Basal cell	1.90 ± 1.16	4.65 ± 1.98	< 0.001
Karyorrhectic cell	0.05 ± 0.02	9.45 ± 1.73	< 0.001
Karyolytic cell	9.60 ± 2.06	85.10 ± 2.69	< 0.001
Cell with Micronucleus	1.50 ± 1.05	12.50 ± 1.43	< 0.001
Total nuclear anomalies (%)	27.25 ± 4.37	152.8 ± 4.75	< 0.001

### The comet analysis technique

The quantitative comet assay parameters for both groups are presented in Table 2. The mean % Tail DNA was significantly higher in cancer patients (55.27 ± 38.13) compared to healthy controls (8.94 ± 6.56), with a highly significant difference (p < 0.001). Similarly, the Olive Tail Moment was significantly elevated in the patient group (48.57 ± 44.89) versus controls (2.11 ± 1.80), p = 0.001. The Tail Length was also significantly greater in cancer patients (27.46 ± 5.90 px) compared to controls (6.31 ± 4.87 px), p < 0.001. These findings indicate substantially higher DNA fragmentation in cancer patients, confirming the genotoxic effects associated with malignancy.

**Table 2.** Comet assay parameters in cancer patients and control group

Parameter	Control Group (mean ± SD)	Patient Group (mean ± SD)	p-value
**% Tail DNA**	8.94 ± 6.56	55.27 ± 38.13	< 0.001
**Olive Tail Moment**	2.11 ± 1.80	48.57 ± 44.89	0.001
**Tail Length (px)**	6.31 ± 4.87	27.46 ± 5.90	< 0.001

Statistical analysis was performed using an independent samples t-test. Values are expressed as mean ± standard deviation (SD).

### Study of some biochemical and hematological parameters in studied group

As shown in Table 3 show a significant increase in the mean levels of urea (50.600 ± 8.259) and creatinine (1.073 ± 0.173), and no significant increase in TSB (0.917±0.143) and HCT (38.080 ± 2.311) in cancer patients compared with the control. Calcium (9.010 ± 0.680), Hb (8.322 ± 1.247), Plt (91.300 ± 24.495), and White Blood Cell count (11.920 ± 2.833) levels were significantly lower in the patient group compared to the control group.

**Table 3.** Mean level of some biochemical and hematological parameters in studied group.

Parameter	Controls (mean ± SD)	Patients (mean ± SD)	p-value (sig)
Urea	31.266 ± 3.278	50.600 ± 8.259	0.001
Creatinine	0.723 ± 0.175	1.073 ± 0.173	0.016
Calcium	8.213 ± 0.262	9.010 ± 0.680	0.001
TSB	0.461 ± 0.080	0.917 ± 0.143	0.072
WBC	4.120 ± 1.007	11.920 ± 2.833	0.057
Hb	12.033 ± 0.849	8.322 ± 1.247	0.063
HCT	29.153 ± 3.341	38.080 ± 2.311	0.306
Platelets	292.300 ± 83.085	91.300 ± 24.495	0.002

## Discussion

Cancer is characterized by genomic instability, which can be detected through various cytogenetic biomarkers. In this study, we evaluated DNA damage in cancer patients using the buccal micronucleus assay and the comet assay, alongside biochemical and hematological parameters.

**Figure 1.**
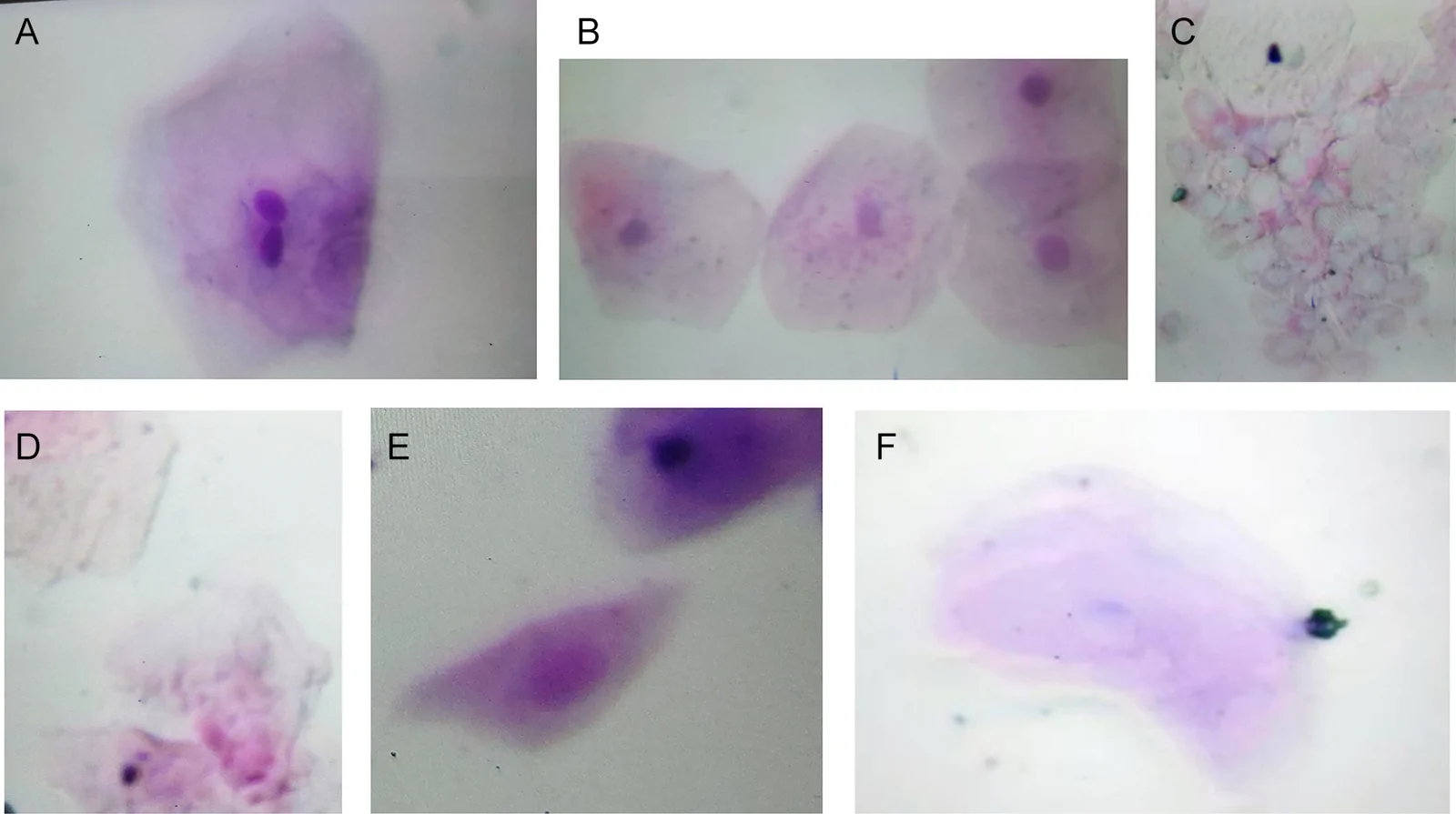
Photomicrograph showing micronuclei (400x). (A) Binucleated cell, (B) normal cell, (C) chemotherapy effects cells, (D) Karyorrhetic cell, (E) Basal cell, (F) Karyolytic cell.

As shown in Table 1 and illustrated in Figure 1, the frequency of micronuclei in the epithelial cells of the patient group was significantly higher in the patient group compared to the control group indicating increased genomic instability in cancer patients.

A cytogenetic method for assessing DNA damage and indications of cell death in oral epithelial cell micronuclei testing. The initial line of defense against ingestion or absorption consists of the epithelial cells of the oral lining. Condensed chromatin and disappearance of karyolitic nuclear protein, which manifests as a ghost, are two signs of cell deterioration. ^16^ Because chromosomal instability is a continuous process rather than a stable condition, such as aneuploidy, it may go undetected by commonly used diagnostic techniques such as sequencing. A fairly reliable indicator of chromosomal instability, the presence of micronuclei, historical markers of DNA damage, is simpler to find in tissues than mitotic figures and can provide a snapshot of the time of continuing mis-segregation. Because of its low cost and minimal invasiveness, the micronucleus assay of peripheral blood cells has been widely employed as a marker of radiation exposure. Because of its high sensitivity, it can also be used as a retrospective dosimeter for ionizing radiation; when a tumor develops, ruptured micronuclei may have more noticeable effects than undamaged micronuclei. Using a marker of micronuclear rupture in biopsies can aid in understanding cancer stages and metastatic risks, as well as predicting the response to immunotherapies, given the significance of catastrophic rupture in initiating immunological responses and genetic rearrangements. ^17^

**Figure 2.**
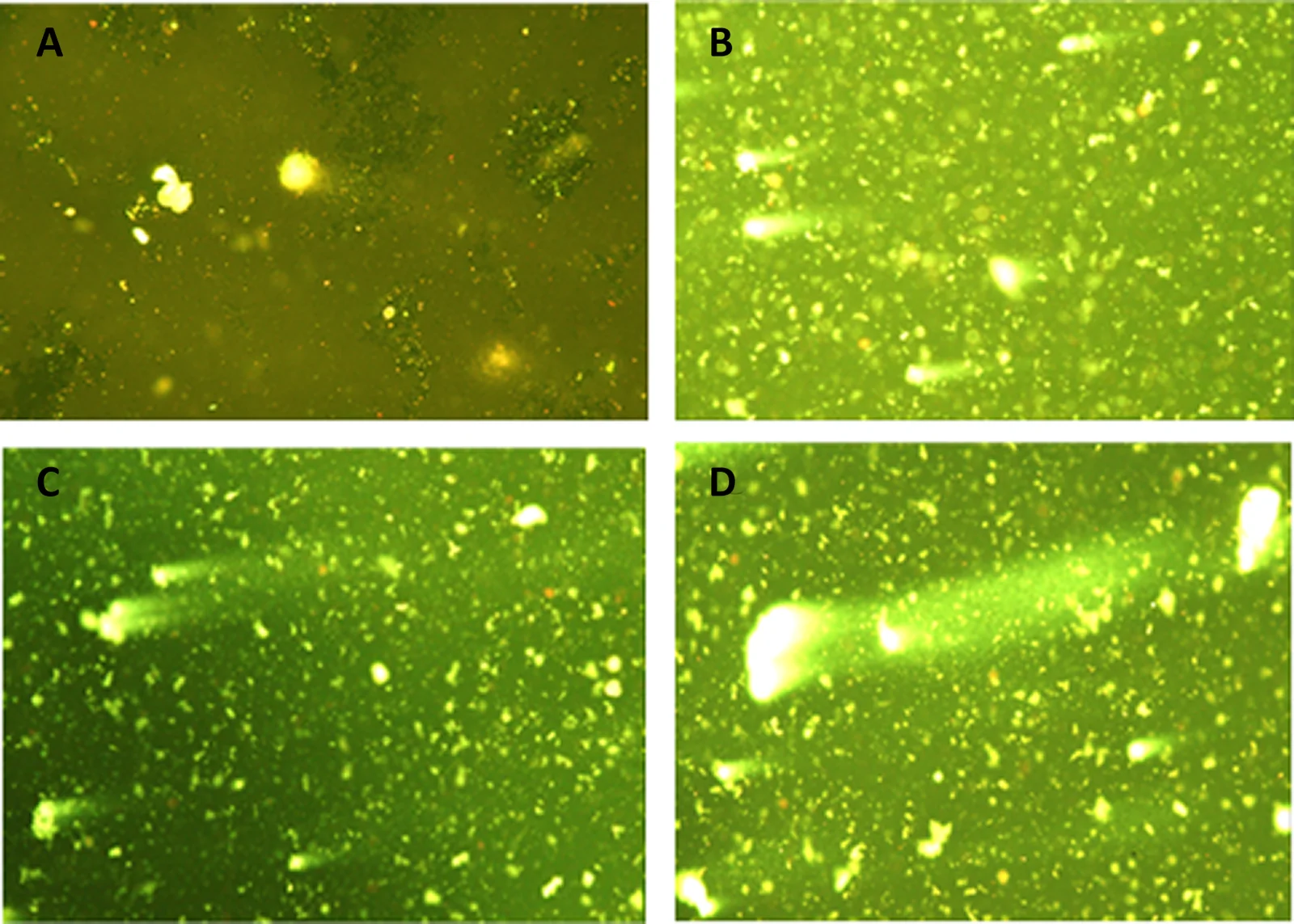
Comet assay images showing DNA damage in lymphocytes from cancer patients and controls. (A) Control group: intact nuclei with no visible tail. (B) Patient group: comet tails indicating DNA fragmentation (magnification 40).

Rarely, some cells may also have nuclear buds or broken eggs, micronuclei (MN) developing next to nuclei in the exact same cytoplasm, or two nuclei in identical cytoplasm (binucleated stage). ^18^ Environmental toxicity can be evaluated by examining these biomarkers when DNA damage and cell death (such as karyolysis or programmed cell death) occur. A novel and exciting method for researching epithelial carcinogens is the use of the mitochondria in shed cells to assess genotoxicity. A sensitive technique for identifying genetic damage in mouth epithelial cells is the examination of their micronuclei ( Figure 2).

Multiple studies have demonstrated that the presence of malignant tumors in an organism causes significant alterations in the chromatin makeup and DNA content of the buccal mucosa. These alterations may function as biochemical indicators of cancerous tumors. ^19^ In individuals with both benign and malignant tumors, some researchers have found changes in the mucous membrane of mouth cells that are connected to malignancy. ^20^

In the present study, micronucleus analysis was performed on buccal mucosal cells (ectodermal origin), while the comet assay was conducted on peripheral blood lymphocytes (mesodermal origin). These two cell types differ in their routes of exposure and biological responses to genotoxic insults. Buccal epithelial cells are directly exposed to mutagens and carcinogens present in inhaled air and ingested food, making them sensitive indicators of local DNA damage. In contrast, lymphocytes reflect systemic exposure to genotoxic agents that enter the bloodstream and reach the bone marrow. The differences observed between the two cell types in our study may be attributed to their distinct embryonic origins, metabolic capacities, and DNA repair mechanisms. This highlights the importance of using multiple tissue types in biomonitoring studies to obtain a comprehensive assessment of genotoxic risk.

Prior research has demonstrated chromosomal abnormalities and an increased incidence of micronuclei in the normal somatic cells of patients with cancer. ^21, 22^ Previous studies found that buccal cells from patients with Alzheimer’s disease, head and neck cancer, and oral cancer had increased micronuclei along with additional nuclear abnormalities. ^23^ The current study discovered raised levels of binucleated and karyolytic cells, among other nuclear abnormalities, which contributed to enhanced miniature nuclei scoring in patients with breast cancer. Cells with two very close major nuclei instead of one are known as binucleated cells, and they are thought to be a biomarker of cytoplasmic preventing cytokinetic defects caused by a condition known as aneuploidy. Karyolitic cells indicate a very late stage of cell death and indicate reactions to cytotoxicity. They appear to have missing nuclei because their nuclear DNA is depleted. ^24^

An increase in tiny nucleated cells among individuals with cancer was studied by Ismail et al. ^9^ They found that exfoliated cells may have altered DNA repair effectiveness and increased genomic instability, which is consistent with recent genotoxic events in dividing cell types and is in line with ongoing research.

In line with other studies of increased levels in buccal cells, Kalender et al. ^25^ found that patients with breast cancer had a higher frequency of micronuclei. Ban et al. ^26^ found that 136 cancer patients had higher MNi frequencies in their lymphocytes than 48 healthy individuals.

In addition to having higher micronuclei scores, cancer patients also had higher levels of a number of nuclear abnormalities, indicating cytotoxicity and genotoxicity, such as compressed chromatin (CC), broken eggs (BE), binucleated cells, karyolysis, and karyorrhexis. A Mexican study of cancer patients revealed similar findings. ^27^

The results we obtained suggest that greater MN along with additional nuclear abnormalities in the removed buccal cells of tissues of cancer patients may be due to genetic instability or alterations in the BRCA1 and BRCA2 genes. One of the primary functions of BRCA proteins is the repair of double-stranded DNA breaks by regulating homologous recombination. Genomic instability caused by inadequate DNA repair increases the MNi in reproductive cells. ^28^

### The comet analysis technique

The comet assay has been used extensively in genotoxicity investigations, bio-monitoring, ecological testing, and human illness research to quantify various cellular responses to DNA damage. In addition to describing the assay’s utility in evaluating oxidative stress within tumors, its potential as a tool for predicting a person’s tumor sensitivity to radiation and other chemotherapeutic medications was investigated.

A comet assay, which involves single-cell electrophoresis on gels, was used to evaluate the extent of DNA impairment in malignant epithelial cells. A DNA-binding dye was used to observe the results under a fluorescence microscope, displaying unique comet-like patterns that indicate the level of genotoxic stress experienced by each individual cell, as illustrated in Figure 2.

The image shows a range of comet morphologies among the studied cells, from intact nuclei with little tail production to those with prominent comet tails. These tails are a sign of DNA strand breaks, because they are created when fragmented DNA migrates during electrophoresis. Heterogeneous levels of DNA destruction within the cell populations are suggested by differences in tail length and intensity.

Cells exhibiting long, diffuse tails with reduced head intensity were considered to have experienced substantial DNA disintegration, which is in line with the genotoxic insult frequently observed in cancerous cells. In contrast, cells with tiny or absent tails were thought to have sustained little damage, possibly indicating more resilient or less damaged subpopulations. ^29^

According to experimental circumstances, this damage pattern could be a result of the cytotoxic effects of external treatments (such as radiation, chemotherapy, or oxidative stress) or the inherent instability of epithelial tissue cancer cells. The results validate the usefulness of the comet assay as a quick and sensitive technique for tracking genotoxic reactions in vitro and identifying Breakage of DNA strands in individual cells. ^30–32^

### Study of some biochemical and hematological parameters in studied group

These results were in agreement with those of Pullakanam et al., ^33^ who showed a decrease in total white blood cells, hemoglobin, and platelets and an increase in HCT in cancer patients compared with controls. Additionally, it concurs with Rimsha et al., ^34^ which showed significant (P < 0.05) variations among cancer patients and control groups in biochemical variables (ALT, AST, and cholesterol) and blood test results (RBCs, H.B., PCV, WBCs, and platelets).

Anemia, a common complication among cancer patients, is frequently detected by measuring hemoglobin and PCV levels; those that, when compared with healthy controls, were typically lower in the sick group. ^35^ Furthermore, these levels are significantly affected by factors including overall clinical health and dietary status. ^36^ Compared to controls, patients’ average corpuscular size and median concentration of corpuscular hemoglobin were significantly lower. ^37^

The discovery that kidney function was diminished in patients with cancer supported the findings of Ibraheem et al., ^38^ who discovered that the levels of serum electrolytes and all kidney function tests were beginning at the upper limit or below the normal range, despite the fact that some levels showed significant variations among patients as well as controls.

**Figure 3.**
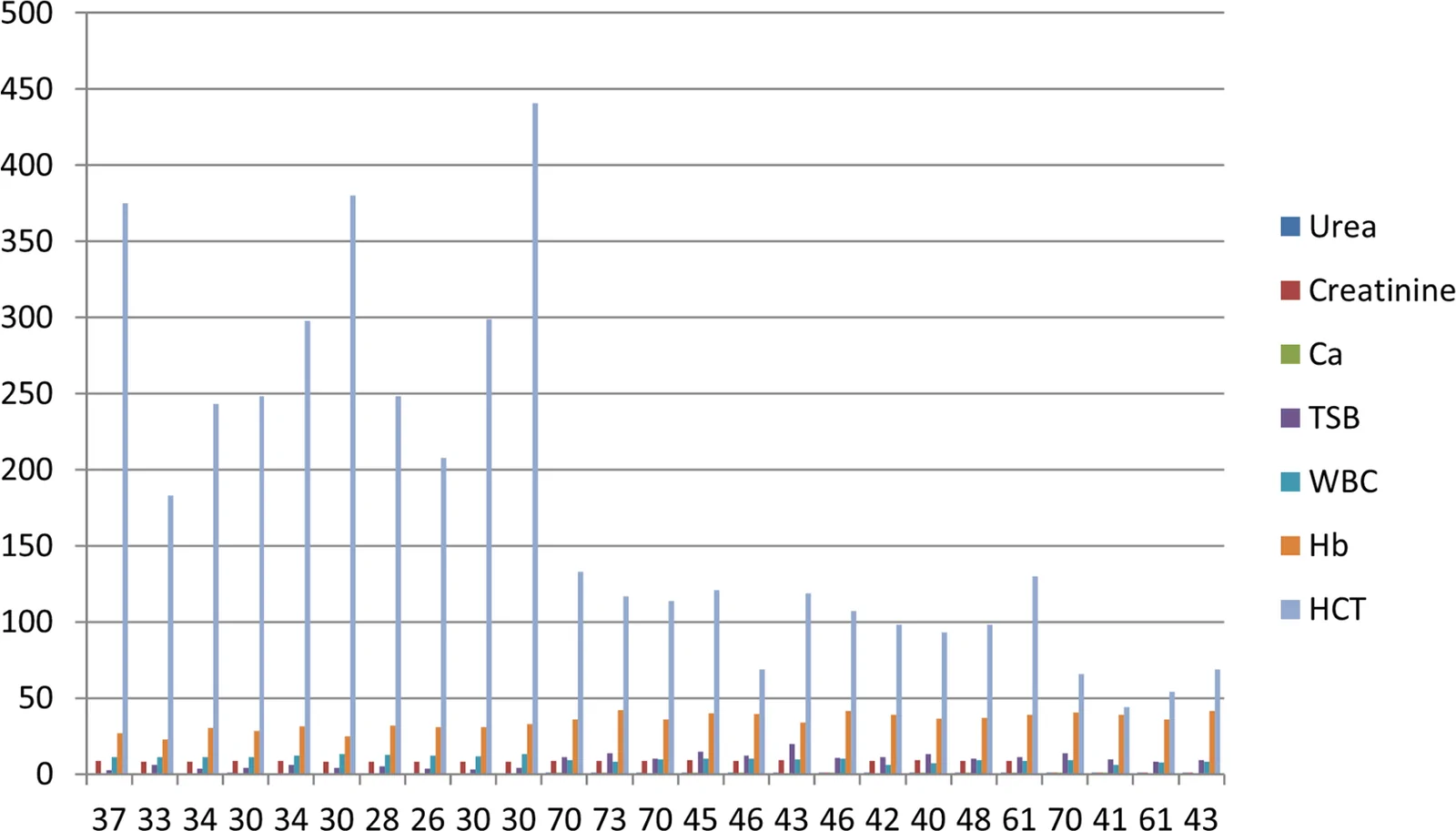
Mean levels of biochemical and hematological parameters (Urea, Creatinine, Ca, TSB, WBC, Hb, HCT) in cancer patients (blue bars) and control group (orange bars). Values are presented as mean ± SD.

Patients with cancer might have other health issues or characteristics that increase the possibility of renal impairment before receiving kidney-toxic therapy. ^39^ Chronic renal disease is prevalent in the elderly, regardless of the presence of cancer. The higher urea levels in the cancer group than in the healthy group may be due to increased protein metabolism following chemotherapy-induced cell death. An independent t-test was performed to compare biochemical and hematological parameters between patients and controls. The results, shown in ( Table 3), indicate statistically significant differences for most parameters, with patients exhibiting higher or lower values than controls. The mean levels of these parameters are visually represented in Figure 3, which illustrates the differences between the studied groups.

One limitation of this study is the absence of multivariate analysis to adjust for confounding factors such as age and smoking, due to the limited sample size. Future studies with larger cohorts are needed to confirm these findings.

## Inclusion

The increased frequency of nuclear anomalies and DNA fragmentation in cancer patients highlights the potential of the buccal MN assay and comet assay as effective, non-invasive tools for early cancer detection and genotoxic monitoring. Alterations in blood and biochemical parameters further support systemic effects of malignancy. Together, these assays may offer a comprehensive approach to cancer screening and evaluation.

## Data Availability

The data supporting the findings of this study are openly available in the Zenodo repository at https://zenodo.org/records/18889026, reference number DOI: 10.5281/zenodo.18889026. ^40^ This dataset contains the SPSS statistical analysis outputs used in the manuscript, including descriptive statistics, tests of homogeneity of variances, ANOVA results, and means plots for the following variables: Urea, Creatinine, Ca, TSB, Hb, WBC, Hct, and plt. The data are aggregated to protect participant confidentiality and do not contain any individual patient information, in accordance with institutional ethics committee guidelines. The dataset is published under the Creative Commons Attribution 4.0 International license (CC-BY 4.0).
