# Supplementary material for: Genotoxicity  effects in Cancer Patients: Evaluation by Micronucleus and Comet Assays and Correlation with Biochemical and Hematological Indices
Source: F1000Res. 2026 Aug 14;15:545. Originally published 2026 Apr 16. [Version 3] doi: 10.12688/f1000research.178233.3 (PMC13562970; doi:10.12688/f1000research.178233.3)
Supplement: Peer review history

# Reviewer report: Reviewer response for version 3

**DOI:** 10.5256/f1000research.208301.r512004

**Published:** 2026-09-10

**Authors:** Paolo Baldo, Valeria Martinello

**Affiliations:** 1 Centro Riferimento Oncologico CRO-Aviano—IRCCS, Aviano, Italy; 2 Riferimento Oncologico IRCCS CRO Aviano, Hospital Pharmacy Centro di, Italy, Italy, Italy

In the Introduction:

*- Carcinoma* *IS*…

- **Cancer is among the leading causes of death worldwide.**
- **Carcinoma is only one category of cancer arising from epithelial tissues.**

- “ *Its classification varies according to the tissue from which it arises and the organs in which the disease develops.”*

**Modern cancer classification depends on:**

- **tissue of origin**
- **histological subtype**
- **molecular characteristics**
- **genetic alterations**
- **immunohistochemical profile**

*- “The type of tumor is determined by microscopic examination of the tumor cells, which is sometimes supported by blood”*

**Diagnosis relies primarily on:**

- **histopathology**
- **immunohistochemistry**
- **molecular pathology**

**Blood tests may provide:**

- **tumor markers**
- **liquid biopsy**
- **circulating tumor DNA**

**but they are generally adjunctive**

*- Authors say: It is usually difficult to determine the cause of the disease; however, we know the general causes of cancer. Many risk factors can cause this disease, such as obesity, lack of physical activity, smoking, and environmental pollution.* *^2^***

*“Although this disease is considered fatal, recovery from it is continually increasing in the majority of cases as a result of improvements in therapeutic options as well as early diagnosis techniques”*

**Not all cancers have improving survival**

**The manuscript repeatedly uses**

**“DNA disintegration”. Better use: DNA damage, DNA fragmentation**

*- “Investigation suggests that buccal cell microscopic nuclei (MNs) are significantly elevated in the buccal mucous membrane cells of people with precancerous tumors and those with cancer, making them possible biomarkers for cancer of the mouth.* *^4^* *Chromosome fragmentation from damaged or disorganized chromosomes or inadequately repaired DNA lesions resulting from mitotic mistakes causes micronuclei to develop in proliferating cells.”*

*“ Deficits in micronutrients are needed as cofactors for DNA disintegration and chromosomal separation,*

**Scientifically incorrect.**

**Micronutrients are not needed for DNA disintegration.**

**Instead, Micronutrient deficiencies impair**

- **DNA synthesis**
- **DNA repair**
- **methylation**
- **antioxidant defenses**

*- "oxidative damage, aneugen exposure to clastogens, genetic anomalies in DNA repair genes and/or cell cycle checkpoints, and more can all contribute to these occurrences.* *^6^* *MN develops as a result of chromosomal rearrangements, modifications in the expression of genes, aneuploidy, and outcomes related to chromosomal instability frequently observed in cancer.”*

*“ Genetic damage is the only factor that the chromosomal aberration test can detect; however mitotic spindle malfunction and chromosome loss due to aneugenic processes can also be detected by the micronucleus test”*

**Chromosomal aberration assays detect:**

- **structural chromosome abnormalities**

**The micronucleus assay detects**

- **chromosome breakage (clastogenicity)**
- **chromosome loss (aneugenicity)**

- In the section: "Participant selection": The manuscript states: " *samples were collected from random people*"

 The authors should describe:

- how participants were recruited
- whether sampling was consecutive or random
- inclusion criteria
- exclusion criteria
- smoking status
- alcohol consumption
- occupational exposure
- medications
- previous chemotherapy or radiotherapy
- chronic diseases

These variables strongly influence micronucleus frequency and comet assay results and are potential confounders **.**

 - In the chapter: *"Alkaline comet assay" *c'è una frase incompleta

*During this test, a treatment technique (Trevigen, Inc., Gaithersburg, US) was applied. The Lysis Solution was prepared and then allowed to cool for 20 min. Add cells with 1 × 10* *^5^* */ml melted low point of melting (LMA) after 50 μl is dispensed onto a Comet SlideTM. Agarose. Agarose and cells were evenly distributed throughout the sample using a pipette tip. After being flattened, the microscope slides were placed in the dark environment and cooled for 10 min at 4 °C. To improve sample adherence in heavy humidity, the gelling time might be prolonged to 30 min.* *After 30 to 60 min, the slides were removed out of a 4 °C Lysis Solution to get rid of any remaining buffer that Com-.* **SENTENCE IS  INCOMPLETE!!!**

 - Poi, in " *The comet analysis technique",*

*".....According to experimental circumstances, this damage pattern could be a result of the cytotoxic effects of external treatments (such as radiation, chemotherapy, or oxidative stress) or the inherent instability of **epithelial tissue cancer cells**."....*

**The manuscript states epithelial tissue cancer cells**

**However, the comet assay was performed on Lymphocytes?**

**Therefore the discussion should refer to peripheral blood lymphocytes, not cancer epithelial cells.**

Is the work clearly and accurately presented and does it cite the current literature?

Yes

If applicable, is the statistical analysis and its interpretation appropriate?

Not applicable

Are all the source data underlying the results available to ensure full reproducibility?

Partly

Is the study design appropriate and is the work technically sound?

Yes

Are the conclusions drawn adequately supported by the results?

Yes

Are sufficient details of methods and analysis provided to allow replication by others?

Partly

Reviewer Expertise:

NA

We confirm that we have read this submission and believe that we have an appropriate level of expertise to confirm that it is of an acceptable scientific standard, however we have significant reservations, as outlined above.

# Reviewer report: Reviewer response for version 2

**DOI:** 10.5256/f1000research.205608.r503793

**Published:** 2026-08-06

**Authors:** Adi Baumgartner

**Affiliations:** 1 York St John University, York, UK

The manuscript by Khalaf and colleagues entitled “Genotoxicity effects in Cancer Patients: Evaluation by Micronucleus and Comet Assays and Correlation with Biochemical and Hematological Indices” uses buccal cell MN and Comet assays in combination with biochemical and hematological endpoints to evaluate genotoxic effects in cancer patients.

 My first impression when reading the manuscript was that this can only be an early draft because of the many errors, typos, fragments of sentences, missing words, misleading and vague statements. The authors are therefore strongly encouraged to carefully amend and correct the text using.

 Worryingly, paraphrasing was pushed too far (possibly by using computer software or AI), e.g. using “issue” instead of “condition” at the beginning of the introduction, “excised” instead of “exfoliated” and “surveillance” instead of “monitoring” in the objectives, “concentrated” instead of “condensed” in the introduction, “relaxation” instead of “unwinding” in the Alkaline Comet assay method paragraph, “miniature” instead of “micro” and “removed” instead of “exfoliated“ both examples on page 9… to name a few.

 What is not clear is whether the individuals in the cancer group were sampled pre-treatment, mid-treatment or post-treatment. The results could then be statistically analyzed by cancer type (which was not done) and by treatment. The authors need to be careful and keep in mind: while the CBMN assay yields MNi in *in-vitro* cultured lymphocytes, MNi in exfoliated buccal cells occur in precursor cells *in vivo* but only postmitotic squamous epithelial cells are evaluated, which do not divide.

 On page 10, reference 37 seems to be out of place and should go to the next paragraph. The authors should check all references in the manuscript.

 Overall, this manuscript will need some work. While the findings are interesting, the writing of the manuscript lacks accuracy and precision. The discussion is weak as it only partly critically discusses the findings in the context of literature, let alone creating an over-arching argument for strongly linking the buccal-lymphocyte-biochemical-hematological findings together (supported by good statistical analysis) and working towards a wholistic picture about the damage cancer can do to normal tissues.

 The comments below contain suggestions to create a better manuscript but do not contain by far all the things that were noticed.

 Specific comments:

 Abstract: Add the plural “s” to “cell”, e.g. basal cells. One fraction of cells is called “broken egg cells” – “broken eggs” means something else. Use “Pyknotic cells” instead of only “condensed chromatin”. The plural of micronucleus is micronuclei – the abbreviation MNi should be used once defined. Typo: It should read “mononucleated cells”. Use frequencies when showing the results: e.g. Karyolytic cells (85.10 ± 2.69 – is it percent or per thousand, whatever was evaluated per patient). The units are missing for the biochemical and hematological results. My strong recommendation: report fold increases plus p-values instead of only findings in the cancer patient group.

 Introduction: Add “analysis” after “supported by blood”. In the 2 ^nd^ paragraph, line 1, define micronuclei as MNi as done further down and not “MNs”, use “micro” instead of “microscopic”. Use “Comet assay” instead of “comet impact assay” in the objectives. Sample preparation needs to go into the Material and method part after how buccal cells were collected (both in one paragraph).

 Methods: What does “washing their salivary glands” mean? Do the authors mean they rinsed their mouth with water? The dye is called “Giemsa”. The Comet assay method description is very confusing, please rewrite; be concise and only describe what is required for reproducibility. Add the field strength V/cm = 21 V divided by the length of the electrophoresis tank the authors used, from electrode to electrode in cm. Have cells been analyzed by double-blinding slides? Have the participants’ samples be anonymized or pseudo-anonymized?

 Results: The units are missing from biochemical and hematological data (also in table 3). Amend the names of the different cell entities in table 1 as suggested above. In table 1, are the values shown as percentages or per 1000 evaluated cells? Add MNi/1000 cells and the MN mean ratio (MR _i_ = MN mean (patients) / MN mean (controls)) to compare better with literature.

 Discussion: Correct “karyolitic” to “karyolytic”. What does “ruptured micronuclei”, “undamaged micronuclei” or “micronuclear rupture” mean? Rephrase. In the 2 ^nd^ paragraph on page 8, you could say “necrosis or apoptosis” instead of “karyolysis or programmed cell death”. In the next line, I assume “mitochondria” should read “micronuclei”. The images in figure 2 are missing size bars. Despite the massive amount of background particles, the cell size in panel D seems huge – could this be an aggregate? Page 9, line 4: the sentence from “cytoplasmic … aneuploidy” is confusing (aneuploidy can cause MNi, but binucleates are caused by defective cytokinesis not aneuploidy – see Holland et al. 2008). What do the authors mean with “tiny nucleated cells” – provide a proper identifiable name. Page 9, 3 ^rd^ paragraph, what does “increased levels of buccal cells” mean? Hyperplasia? Or is this a fragment and a word missing? Provide references at the start of the comet analysis paragraph as a lot of information is provided without providing sources.

 Using the term “malignant epithelial cells” could be misleading as the buccal cells the authors investigated were not cancerous. The tail length and intensity are mostly caused by single-strand breaks and alkaline labile sites; the general term DNA damage should be used – I would not call it DNA destruction; and yes, some lethal damage can lead to cell death but then the nucleus is mostly degraded. Figure 3 is missing appropriate annotations and is confusing. There are no units on the y-axis and there is no indication what the numbers on the x-axis represent; then, in the legend it says patients in blue and controls in orange, which are the same colors also represent urea and Hb. Please amend figure 3. The “Inclusion” paragraph should be called “Conclusion”.

Is the work clearly and accurately presented and does it cite the current literature?

Partly

If applicable, is the statistical analysis and its interpretation appropriate?

Yes

Are all the source data underlying the results available to ensure full reproducibility?

Partly

Is the study design appropriate and is the work technically sound?

Yes

Are the conclusions drawn adequately supported by the results?

Yes

Are sufficient details of methods and analysis provided to allow replication by others?

Partly

Reviewer Expertise:

genotoxicology, oxidative stress, mutagenesis, cancer

I confirm that I have read this submission and believe that I have an appropriate level of expertise to confirm that it is of an acceptable scientific standard, however I have significant reservations, as outlined above.

# Response

**Published:** 2026-08-08

**Authors:** sarab dalaf

**Affiliations:** biology, Not Applicable, Tikrit, Iraq

Response to Reviewer 2

 Dear Reviewer,

 We sincerely thank you for your valuable and detailed comments. We have carefully revised the manuscript based on all your suggestions. Below is a summary of our responses:

 1. Language and terminology: All incorrect terms (e.g., issue, excised, surveillance, relaxation, miniature) have been corrected to proper scientific terminology.

 2. Nuclear abnormalities: We have changed broken eggs to pyknotic cells, corrected plural forms, and clarified that values are expressed per 1000 cells in the abstract, tables, and discussion.

 3. Abstract: We added fold increases for all significant parameters (e.g., binucleated cells ~6.8-fold, karyorrhectic cells ~189-fold) alongside p-values.

 4. Methods: We corrected the staining dye name to Giemsa, clarified the mouth-rinsing procedure, added the electrophoresis field strength (V/cm), and confirmed double-blinding and anonymization.

 5. Discussion: We corrected karyolitic to karyolytic, replaced malignant epithelial cells with exfoliated buccal cells, revised the statement about binucleated cells to indicate defective cytokinesis (not aneuploidy), changed ruptured micronuclei to disrupted micronuclei, mitochondria to micronuclei, and DNA destruction to DNA damage.

 6. References: We moved reference 37 to its correct place in the kidney function paragraph and added reference 41 (Olive & Banáth, 2006) to the Comet assay discussion.

 7. Figures: We added a scale bar = 10 µm to Figure 2 and clarified the axes in Figure 3 with appropriate units and labels.

 8. Section title: We changed Inclusion to Conclusion.

 We believe the manuscript is now significantly improved and hope it meets your expectations.

 Kind regards,

 Sarab Dalaf Khalaf (Corresponding Author)

 Department of Biology, College of Science, Tikrit University

 Email: sarab.dalaf@tu.edu.iq

# Reviewer report: Reviewer response for version 2

**DOI:** 10.5256/f1000research.205608.r502246

**Published:** 2026-07-25

**Authors:** Ayse Gul Zamani, Nihan Somuncu

**Affiliations:** 1 Medical Genetics, Necmettin Erbakan University Meram Faculty of Medicine (Ringgold ID: 427826), Meram, Konya, Turkey; 2 Neccmettin ERbakan University, Konya, Konya, Turkey

Authors have carefully considered the suggestions and made the requested changes. The changes made are in line with my suggestions. This article is acceptable in its current form.

Is the work clearly and accurately presented and does it cite the current literature?

Yes

If applicable, is the statistical analysis and its interpretation appropriate?

Yes

Are all the source data underlying the results available to ensure full reproducibility?

Partly

Is the study design appropriate and is the work technically sound?

Yes

Are the conclusions drawn adequately supported by the results?

Partly

Are sufficient details of methods and analysis provided to allow replication by others?

Partly

Reviewer Expertise:

Medical genetics, molecular genetics, cancer

We confirm that we have read this submission and believe that we have an appropriate level of expertise to confirm that it is of an acceptable scientific standard.

# Reviewer report: Reviewer response for version 1

**DOI:** 10.5256/f1000research.196600.r496337

**Published:** 2026-07-02

**Authors:** Ayse Gul Zamani, Nihan Somuncu

**Affiliations:** 1 Medical Genetics, Necmettin Erbakan University Meram Faculty of Medicine (Ringgold ID: 427826), Meram, Konya, Turkey; 2 Neccmettin ERbakan University, Konya, Konya, Turkey

**This is a well-planned and meticulously researched work. However, there are sections that need correction, addition, and discussion.**

**Below are the points that caught my attention while evaluating the article.**

  

- In human micronucleus (MN) studies (especially in peripheral blood lymphocytes or cheek epithelial cells), using a standardized questionnaire aimed at collecting information about lifestyle and environmental factors is an international requirement. This is because micronucleus frequency (DNA damage) is affected by almost everything, from age and diet to smoking and X-rays. Did you use such a questionnaire? If so, mentioning the questionnaire you used in the methodology section of your study will directly increase the prestige and reliability of your study.

If you only compare your patient and control groups as "patient vs. healthy," reviewers will rightly ask questions such as: "What if the number of smokers in the patient group is much higher than in the control group, and the damage is actually caused by smoking?"

 To avoid this, you should include the data you obtained from the questionnaire in Multiple Linear Regression or ANCOVA (Analysis of Covariance) tests in your statistical software (SPSS, GraphPad Prism). These tests allow you to mathematically "neutralize" (adjust for confounders) the effects of confounding factors such as age, smoking, and diet, and prove the true, pure effect of disease (cancer) on DNA damage.

 There are many general and dental studies in the literature regarding the micronucleus and comet methods. Dental studies have aimed to evaluate the effects of chemical agents, infections, and oral cancers. Were other oral factors excluded in this study? If so, this should be stated in the study.

- The materials and methods section includes a heading "The Comet Analysis Technique." However, the procedures performed under this heading are not mentioned. It may have been overlooked. The parameters that should be compared when evaluating the comet test results of the control and patient groups are: % Tail DNA, Olive Tail Moment (OTM), and Tail Length. The article does not include a comparison result for these parameters. There is no relevant table. These results should be added to the study and discussed based on these findings.  
- Histopathological diagnoses of cancer patients should be stated.
- Were these samples taken before chemotherapy?    
- The discussion section should not begin with an explanation of Table and Figure 1. I believe this section should be incorporated into the text of the next paragraph.
-  In the discussion section, MN analyses performed on exfoliative cells and comet tests from peripheral blood should be examined in more detail. The origin of cells from different germ layers (ectoderm and mesoderm) can lead to different responses to environmental and lifestyle factors (e.g., cigarette smoke, inhaled chemicals, or systemic drugs) and cancer development. Oral cells (ectoderm): They are directly exposed to mutagens inhaled and ingested through the respiratory and digestive tracts. Blood cells (mesoderm): They better reflect toxic substances that enter the bloodstream after entering the body, are distributed systemically, and cause damage in the bone marrow. Therefore, changes in blood cell data, in particular, can be highlighted in terms of cancer. The effect of cancer on cells of two different origins can be discussed.  
- To which group do the values ​​given in Figure 3 belong? Are the data for both groups presented together? This figure was not considered to be explanatory or contributing to the study.

Is the work clearly and accurately presented and does it cite the current literature?

Yes

If applicable, is the statistical analysis and its interpretation appropriate?

Yes

Are all the source data underlying the results available to ensure full reproducibility?

Partly

Is the study design appropriate and is the work technically sound?

Yes

Are the conclusions drawn adequately supported by the results?

Partly

Are sufficient details of methods and analysis provided to allow replication by others?

Partly

Reviewer Expertise:

Medical genetics, molecular genetics, cancer

We confirm that we have read this submission and believe that we have an appropriate level of expertise to confirm that it is of an acceptable scientific standard, however we have significant reservations, as outlined above.

# Response

**Published:** 2026-07-03

**Authors:** sarab dalaf

**Affiliations:** biology, Not Applicable, Tikrit, Iraq

Response to Reviewer (Approved with Reservations)

 Dear Reviewer,

 We sincerely thank you for your valuable time and constructive comments on our manuscript entitled "Genotoxicity effects in Cancer Patients: Evaluation by Micronucleus and Comet Assays and Correlation with Biochemical and Hematological Indices". We have carefully considered all your observations and have revised the manuscript accordingly in the version posted on F1000Research. Below, we provide our point-by-point responses to each of your comments. All changes have been highlighted in the revised manuscript.

 Reviewer Comment 1: Regarding the use of a standardized questionnaire and statistical adjustment for confounding factors (age, smoking, diet).

 Response: We thank the reviewer for this important comment. We confirm that a questionnaire was administered to collect data on age, smoking, and lifestyle factors from all participants. However, due to the limited sample size, we did not perform multiple linear regression or ANCOVA analysis. We have now added this limitation in the discussion section (Page 8) and will address this in future studies with larger cohorts.

 Reviewer Comment 2: Regarding Figure 3, to which group do the values belong?

 Response: We apologize for this omission. We have now added a clear legend to Figure 3 indicating that blue bars represent cancer patients and orange bars represent the control group. The revised figure legend now reads: "Figure 3. Mean levels of biochemical and hematological parameters (Urea, Creatinine, Ca, TSB, WBC, Hb, HCT) in cancer patients (blue bars) and control group (orange bars). Values are presented as mean ± SD."

 Reviewer Comment 3: Regarding the missing comet assay parameters (% Tail DNA, Olive Tail Moment, and Tail Length).

 Response: We thank the reviewer for this valuable observation. We have now added a new table (Table 3) in the Results section presenting the comet assay parameters (% Tail DNA, Olive Tail Moment, and Tail Length) for both patient and control groups, along with the statistical significance. The quantitative analysis revealed significantly higher values in cancer patients compared to controls for all three parameters (p < 0.001 for % Tail DNA and Tail Length; p = 0.001 for Olive Tail Moment). This table is now referenced in the text.

 Reviewer Comment 4: Regarding the beginning of the Discussion section.

 Response: We agree with the reviewer. The Discussion section has been revised to begin with a general background on genomic instability in cancer, and the explanation of Table 1 and Figure 1 has been moved to the second paragraph.

 Reviewer Comment 5: Regarding the discussion of cell origin differences (ectoderm vs mesoderm).

 Response: We thank the reviewer for this insightful suggestion. We have added a new paragraph in the Discussion section addressing the differences between buccal mucosal cells (ectodermal origin) used for the micronucleus assay and peripheral blood lymphocytes (mesodermal origin) used for the comet assay. We discuss the distinct exposure routes, biological responses, and the importance of using multiple tissue types in biomonitoring studies.

 Reviewer Comment 6: Regarding histopathological diagnoses and whether samples were taken before chemotherapy.

 Response: We appreciate this comment. We have now added a statement in the Materials and Methods section clarifying that all samples were collected from cancer patients prior to the initiation of any chemotherapy or radiotherapy, and that histopathological diagnoses were confirmed by the oncology center for each patient.

 We hope that our responses and revisions adequately address all your concerns and meet your expectations. Thank you again for your valuable input, which has significantly improved the quality of our work.

 Sarab Dalaf Khalaf (Corresponding Author)

 Department of Biology, College of Science, Tikrit University

 Email: sarab.dalaf@tu.edu.iq

# Response

**Published:** 2026-07-04

**Authors:** sarab dalaf

**Affiliations:** biology, Not Applicable, Tikrit, Iraq

Clarification regarding Comment 6:

 Upon re-verification, we found that treatment timing was not documented for all patients. Therefore, this statement was not added to the manuscript, and we acknowledge this as a limitation. We apologize for any confusion.
